# Neurological and neuropsychiatric disorders associated with COVID-19. Part I: overview and neurological disorders

**DOI:** 10.31744/einstein_journal/2021CE6448

**Published:** 2021-10-20

**Authors:** Martina Giacalone, Marcos Roberto Tovani-Palone, Luca Marin, Massimiliano Febbi, Tommaso Russano, Andrea Giacalone

**Affiliations:** 1 Faculty of Medicine and Pharmacy, Medicine and Surgery Sapienza University of Rome Rome Italy Faculty of Medicine and Pharmacy, Medicine and Surgery, Sapienza University of Rome, Rome, Italy.; 2 Faculdade de Medicina de Ribeirão Preto Universidade de São Paulo Ribeirão PretoSP Brazil Faculdade de Medicina de Ribeirão Preto, Universidade de São Paulo, Ribeirão Preto, SP, Brazil.; 3 Department of Rehabilitation Faculty of Medicine University of Ostrava Ostrava Czech Republic Department of Rehabilitation, Faculty of Medicine, University of Ostrava, Ostrava, Czech Republic.; 4 Faculty of Medicine and Psychology Sant’Andrea Hospital Sapienza University of Rome Rome Italy Department of Neuroscience, Mental Health and Sensory Organs (NESMOS), Faculty of Medicine and Psychology, Sant’Andrea Hospital, Sapienza University of Rome,Rome, Italy.; 5 Department of Industrial Engineering, Technologies for Sports Medicine and Rehabilitation University of Rome Tor Vergata Rome Italy Department of Industrial Engineering, Technologies for Sports Medicine and Rehabilitation, University of Rome Tor Vergata, Rome, Italy.

Dear Editor,

Coronavirus disease 2019 (COVID-19) is an emerging infectious disease caused by severe acute respiratory syndrome coronavirus 2 (SARS-CoV-2), which is a highly contagious and pathogenic virus. In December 2019, the World Health Organization (WHO) was notified of a cluster of atypical pneumonia cases in Wuhan, China. A few weeks later, in January 2020, Chinese authorities determined that the outbreak was caused by a new coronavirus. To date, the total number of confirmed COVID-19 cases worldwide exceeds 200 million, and more than 4.4 million people have died from the disease.^(1)^

The changes in global health status caused by the COVID-19 pandemic have been a matter of great concern with a focus on the numerous clinical manifestations of the disease. In this scenario, an increasing number of studies have highlighted the neuroinvasive potential of COVID-19 and the possible manifestations of the SARS-CoV-2 invasion in the central (CNS) and peripheral nervous systems (PNS).^(2)^ In addition to the wide range of physical disabilities caused by COVID-19, this disease also represents a risk for the mental health of the millions of affected people.^(3)^

We discuss in two letters the most recent findings regarding neurological and neuropsychiatric disorders associated with COVID-19.

## Central nervous system involvement in COVID-19 patients

In the context of COVID-19, many questions about the nervous system involvement remain unanswered. Considering this, several researches have been conducted worldwide on this subject. Current evidence suggests that the CNS invasion may be linked to the viremic spread of SARS-CoV-2, which enables the virus to reach the cerebral circulation and break through the blood-brain-barrier. Similarly to what happens at the airway level, the virus can gain access to the brain by binding to membrane-bound angiotensin-converting enzyme 2 (ACE2), expressed on the endothelium, through the viral S protein spike. The ACE2 expression in neurons and glial cells sheds light on new potential sites of viral invasion. In addition to the hematogenous route, a local spread of the virus from the nasal mucosa and through the cribriform plate has been suggested. The pathophysiology of most CNS manifestations seems to be related primarily to the cytokine storm induced by the viral infection, as well as by the ensuing cerebral edema and neuronal apoptosis.^(4)^ The most common neurological symptoms observed so far in these patients include headache, dizziness, myalgia, fatigue, and consciousness alterations.^(5,6)^ The pathogenic mechanisms underlying consciousness alteration are difficult to define; however, this sign could be explained by the effect of the systemic inflammation due to COVID-19.^(7)^

It is worth mentioning that acute cerebrovascular diseases, such as ischemic and hemorrhagic strokes, have been described by neurologists in COVID-19 patients. These acute neurological events have been observed with a higher incidence in patients with severe coagulopathies. Older patients seem to be at increased risk for such acute events. Indeed, they are more likely to have cerebrovascular risk factors, which together with the coagulopathy caused by the viral infection and the related inflammation, make them more susceptible to develop acute cerebrovascular events.^(8)^

The relationship of vascular endothelial dysfunction and coagulopathy with severe complications of COVID-19 is supported by evidence of increased levels of D-dimers and pro-coagulant factors, such as fibrinogen.^(9,10)^ Routine prophylactic heparin administration has been shown to significantly reduce mortality in patients with severe coagulation disorders.^(9,11)^

Although prophylactic anticoagulation appears to be a good strategy for the prevention of ischemic stroke in patients with high levels of D-dimer, the management of hypertensive patients at risk of intracranial hemorrhage is still quite challenging and more studies are needed to better define the principles for such management.^(4)^

Another relevant point is that some case reports have highlighted encephalitis, meningitis, and seizures as potential consequences of SARS-CoV-2 infection, supported by evidence of viral traces in the liquor of some patients.^(12,13)^ However, many of the existing studies on this topic report incomplete clinical and treatment data. Moreover, cases of disseminated acute encephalomyelitis have also been described in SARS-CoV-2 affected patients. In this group, the demyelination was observed weeks after the infection.^(14)^ Additionally, a case of myelitis in a patient with SARS-CoV-2 infection was reported.^(15)^ In view of the growing number of COVID-19 cases, it must be of central importance to health professionals who treat COVID-19 patients to assess and investigate potential neurological changes that may be related to the disease. This would help to better understand the neurological manifestations of COVID-19, which are undoubtedly less frequent compared to other symptoms of the disease, but which can nevertheless cause serious sequelae.

## Peripheral nervous system involvement in COVID-19 patients

Results of new research and reports have led several scientists to investigate whether COVID-19 affects the PNS, as well as its effects at the CNS level, and related manifestations. Among the symptoms that can be associated with PNS involvement, it is important to mention that myalgia and muscle fatigue have been reported in many COVID-19 patients. Their occurrence has been associated with a significant increase in the creatine levels.^(16)^ A pioneering study further investigated changes in smell, taste, and chemesthetic function before and after viral infection. The results suggest that changes in chemesthetic perception may be related to changes in sensory neurons in response to SARS-CoV-2 infection. These virus-induced neuronal changes appear to be of a neuropathic nature and mediated by ACE2 receptors.^(17)^

Interestingly, some COVID-19 patients have been also diagnosed with Guillain-Barré syndrome.^(18)^ Considering the number of COVID-19 confirmed cases worldwide, the incidence of association with Guillain-Barré syndrome has not been particularly high.^(19)^A para-infectious profile has been described in these patients instead of the most usual post-infectious profile. A variant of Guillain-Barré syndrome, the Miller Fisher syndrome, has also been described in COVID-19 patients.^(20)^ However, from a microbiological perspective, the understanding of Guillain-Barré syndrome in the setting of COVID-19 is limited due to the lack of adequate test upon admission.^(21)^

Despite Guillain-Barré syndrome appearing to be associated with SARS-CoV-2 infection, the small number of published studies and the difficulty in demonstrating an unequivocal causal link makes this hypothesis only speculative.
