# Supplementary material for: Neurological and neuropsychiatric disorders associated with COVID-19. Part I: overview and neurological disorders
Source: Einstein (Sao Paulo). 2021 Oct 20;19:eCE6448. doi: 10.31744/einstein_journal/2021CE6448 (PMC8528447; doi:10.31744/einstein_journal/2021CE6448)
Supplement: Translation

# Translation: Distúrbios neurológicos e neuropsiquiátricos associados à COVID-19. Parte I: visão geral e distúrbios neurológicos

**Authors:** Martina Giacalone, Marcos Roberto Tovani-Palone, Luca Marin, Massimiliano Febbi, Tommaso Russano, Andrea Giacalone

**Affiliations:** 1 Italy Faculty of Medicine and Pharmacy, Medicine and Surgery, Sapienza University of Rome, Rome, Italy.; 2 Brasil Faculdade de Medicina de Ribeirão Preto, Universidade de São Paulo, Ribeirão Preto, SP, Brasil.; 3 Czech Republic Department of Rehabilitation, Faculty of Medicine, University of Ostrava, Ostrava, Czech Republic.; 4 Italy Department of Neuroscience, Mental Health and Sensory Organs (NESMOS), Faculty of Medicine and Psychology, Sant’Andrea Hospital, Sapienza University of Rome, Rome, Italy.; 5 Italy Department of Industrial Engineering, Technologies for Sports Medicine and Rehabilitation, University of Rome Tor Vergata, Rome, Italy.

Caro Editor,

A doença pelo coronavírus 2019 (COVID-19) é uma doença infecciosa emergente causada pelo coronavírus da síndrome respiratória aguda grave 2 (SARS-CoV-2), o qual corresponde a um vírus altamente contagioso e patogênico. Em dezembro de 2019, a Organização Mundial da Saúde (OMS) foi notificada sobre a ocorrência de um surto de casos de pneumonia atípica em Wuhan, na China. Algumas semanas depois, em janeiro de 2020, as autoridades chinesas determinaram que o surto foi causado por um novo coronavírus. Até o momento, o número total de casos de COVID-19 em todo o mundo ultrapassa os 200 milhões, sendo que mais de 4,4 milhões de pessoas já morreram por conta da doença.^(1)^

As mudanças no estado de saúde global devido à pandemia da COVID-19 têm sido motivo de grande preocupação, com ênfase nas diferentes manifestações clínicas da doença. Nesse cenário, um número crescente de estudos tem destacado o potencial neuroinvasivo da COVID-19, bem como as possíveis manifestações decorrentes da invasão do SARS-CoV-2 no sistema nervoso central (SNC) e periférico (SNP).^(2)^ Além da grande variedade de disfunções físicas causadas pela COVID-19, a doença também representa um risco para a saúde mental das milhares de pessoas infectadas.^(3)^

Nós discutimos em duas cartas os achados mais recentes sobre os distúrbios neurológicos e neuropsiquiátricos associados à COVID-19.

## Envolvimento do sistema nervoso central em pacientes com COVID-19

No contexto da COVID-19, muitos questionamentos sobre o envolvimento do sistema nervoso permanecem ainda sem respostas. Diante disso, diversas pesquisas têm sido conduzidas em todo o mundo sobre o assunto. Evidências atuais sugerem que a disseminação virêmica do SARS-CoV-2 deve estar relacionada com a invasão do SNC, de modo a possibilitar que o vírus atinja a circulação cerebral e atravesse a barreira hematoencefálica. Da mesma forma como ocorre no nível das vias aéreas, o vírus pode alcançar o cérebro, ligando-se à enzima conversora da angiotensina 2 (ACE2), expressa no endotélio, por meio da proteína viral *spike*. A expressão da ACE2 em neurônios e células gliais lança luz sobre novos possíveis locais de invasão viral. Além da via hematogênica, uma difusão local do vírus, a partir da mucosa nasal e através da placa cribiforme, também tem sido sugerida. A fisiopatologia da maioria das manifestações do SNC parece estar relacionada principalmente à tempestade de citocinas induzida pela infecção viral, bem como pelo edema cerebral e a apoptose neuronal subsequentes.^(4)^ Atualmente, os sintomas neurológicos mais comuns observados nesses pacientes incluem cefaleia, tontura, mialgia, fadiga e alterações da consciência.^(5,6)^ Os mecanismos patogênicos subjacentes à alteração da consciência são difíceis de serem determinados, contudo a ocorrência desse sinal pode ser explicada pelo efeito da inflamação sistêmica causada pela COVID-19.^(7)^

Vale ressaltar que a ocorrência de doenças cerebrovasculares agudas, como acidente vascular cerebral (AVC) isquêmico e hemorrágico, tem sido reportada por neurologistas em pacientes com COVID-19. Esses eventos neurológicos agudos têm sido observados com maior incidência em pacientes com coagulopatias graves. Pacientes idosos parecem apresentar maior risco para a ocorrência de tais eventos agudos. De fato, eles são mais propensos a apresentar fatores de risco cerebrovasculares, os quais, juntamente da coagulopatia causada pela infecção viral e da inflamação relacionada, os tornam mais suscetíveis a desenvolver eventos agudos cerebrovasculares.^(8)^

A relação existente entre disfunção vascular endotelial e coagulopatia diante de complicações graves da COVID-19 é suportada pela evidência de níveis aumentados de dímeros-D e fatores pró-coagulantes, como fibrinogênio.^(9,10)^ A administração profilática de rotina com heparina tem sido mostrada como importante aliada na redução da mortalidade em pacientes com distúrbios graves de coagulação.^(9,11)^

Embora a anticoagulação profilática pareça ser uma boa estratégia para a prevenção de AVC isquêmico em pacientes com altos níveis de dímeros-D, o manejo de pacientes hipertensos com risco de hemorragia intracraniana é ainda bastante desafiador, e mais estudos são necessários para melhor definir os princípios para esse manejo.^(4)^

Outro ponto relevante é que alguns relatos de casos têm destacado a ocorrência de encefalite, meningite e convulsões como possíveis consequências da infecção por SARS-CoV-2 – nesse caso, com base na evidência da presença de traços virais no líquor de pacientes com COVID-19.^(12,13)^ Todavia, muitos dos estudos de publicações sobre o assunto demonstram o uso de dados clínicos e de tratamento incompletos. Além disso, casos de encefalomielite aguda disseminada têm sido descritos em pacientes infectados por SARS-CoV-2. Nesse grupo, a forma desmielinizante geralmente ocorre semanas após a infecção.^(14)^ Também, um caso de mielite foi reportado em um paciente com infecção por SARS-CoV-2.^(15)^ Diante do aumento no número de casos de COVID-19, é de suma importância, para profissionais de saúde que prestam assistência a pacientes com essa doença, que realizem a avaliação e a investigação das possíveis alterações neurológicas associadas. Isso deve ajudar a melhor entender as manifestações neurológicas da COVID-19, que são, sem dúvida, menos frequentes em comparação com outros sintomas da doença, mas que podem causar sequelas graves.

## Envolvimento do sistema nervoso periférico em pacientes com COVID-19

Resultados de pesquisas recentes e relatos têm levado vários cientistas a investigar se a COVID-19 causa danos ao SNP, bem como seus efeitos a nível do SNC, além de manifestações relacionadas. Dentre os sintomas que podem estar associados com o envolvimento do SNP, vale mencionar que a mialgia e a fadiga muscular têm sido reportadas em muitos dos pacientes com COVID-19. A ocorrência desses sintomas tem sido associada com aumento significativo dos níveis de creatina.^(16)^ Um estudo pioneiro investigou ainda as alterações de olfato, paladar e das sensações quimestésicas antes e depois da infecção viral. Os resultados encontrados sugerem que mudanças na percepção quimestésica podem estar relacionadas a alterações nos neurônios sensoriais em resposta à infecção por SARS-CoV-2. Tais alterações neuronais induzidas pelo vírus parecem ser de natureza neuropática e mediadas por receptores ACE2.^(17)^

Curiosamente, alguns pacientes com COVID-19 têm também sido diagnosticados com síndrome de Guillain-Barré.^(18)^ Considerando o número de casos confirmados de COVID-19 em todo o mundo, a incidência da associação com a síndrome de Guillain-Barré não tem sido particularmente alta.^(19)^ Um perfil parainfeccioso tem sido descrito nesses pacientes, ao invés do perfil pós-infeccioso mais comum. A síndrome de Miller-Fisher, uma variante da síndrome de Guillain-Barré, também tem sido reportada em indivíduos com COVID-19.^(20)^ Entretanto, o entendimento sobre a ocorrência da síndrome de Guillain-Barré no contexto da COVID-19 é ainda limitado, do ponto de vista microbiológico, devido à falta de testagem adequada na admissão dos pacientes.^(21)^

Além do mais, apesar da existência de indícios de associação da síndrome de Guillain-Barré com a infecção por SARS-CoV-2, a escassez de estudos publicados e a dificuldade em se demonstrar uma ligação causal inequívoca tornam essa hipótese ainda apenas especulativa.
